# Design of 3D printable prosthetic foot to implement nonlinear stiffness behavior of human toe joint based on finite element analysis

**DOI:** 10.1038/s41598-021-98839-3

**Published:** 2021-10-05

**Authors:** Hui-Jin Um, Heon-Su Kim, Woolim Hong, Hak-Sung Kim, Pilwon Hur

**Affiliations:** 1 Department of Mechanical Engineering, Hanyang University, Seoul, South Korea; 2 J. Mike Walker ’66 Department of Mechanical Engineering, Texas A&M University, College Station, TX 77843 USA; 3 Institute of Nano Science and Technology, Hanyang University, Seoul, South Korea; 4 School of Mechanical Engineering, Gwangju Institute of Science and Technology, Gwangju, 61005 South Korea

**Keywords:** Mechanical engineering, Biomedical engineering

## Abstract

Toe joint is known as one of the critical factors in designing a prosthetic foot due to its nonlinear stiffness characteristic. This stiffness characteristic provides a general feeling of springiness in the toe-off and it also affects the ankle kinetics. In this study, the toe part of the prosthetic foot was designed to improve walking performance. The toe joint was implemented as a single part suitable for 3D printing. The various shape factors such as curved shape, bending space, auxetic structure, and bending zone were applied to mimic human foot characteristics. The finite element analysis (FEA) was conducted to simulate terminal stance (from heel-off to toe-off) using the designed prosthetic foot. To find the structure with characteristics similar to the human foot, the optimization was performed based on the toe joint geometries. As a result, the optimized foot showed good agreement with human foot behavior in the toe torque-angle curve. Finally, the simulation conditions were validated by comparing with human walking data and it was confirmed that the designed prosthetic foot structure can implement the human foot function.

## Introduction

Worldwide, over hundred thousand people lose their limbs due to diseases such as cancer and diabetes, as well as violence and car accidents each year^1^. In the United States, approximately 600,000 individuals had major lower-limb amputation in 2005, and this number is estimated to reach twice by 2050^2^. Therefore, the design of prosthetic foot has been widely studied for the lower extremity amputees in the past years. The common goal of prosthetic foot design is to mimic the human gait as closely as possible without causing discomfort to the user^3,4^. The earliest prosthetic foot model is a solid ankle cushioning heel (SACH)^5,6^. The SACH foot consists of a wedge and an internal keel without ankle articulation. Although the SACH foot allows the forefoot to flex, it has several disadvantages due to inflexible rigid keel: poor shock absorption, poor energy release, and user discomfort. To solve these problems, many researchers have developed articulated foot or energy storing/releasing foot that can generate a more human-like foot motion^7–9^. Specifically, the powered prosthetic foot capable of controlling ankle joint stiffness is widely studied^8,9^. Compared to the ankle joint, however, the toe joint has less highlighted to achieve a human foot characteristic for the prosthesis. To date, the conventionally developed prosthetic feet usually consisted of a single part in the forefoot, which is far from the segmented human foot with toe joint. The toe joint is one of the critical factors in designing a prosthetic foot since the toe joint stiffness affects the ankle kinetics and provides a general feeling of springiness during toe-off^10,11^. It is because the toe joint modulates the stiffness by changing the foot arches, which is called the windlass mechanism^12,13^. By this mechanism, the foot becomes elastic at the heel-off, providing a natural foot-rolling and a load transfer, and stiff at the toe-off, helping the kicking motion. Therefore, it is important to understand how the toe joint affects gait behavior and how to implement it. Several attempts were made to investigate the toe joint in the prosthetic foot design^10–15^. Zhu et al. developed the prosthetic foot with the powered ankle and toe joint to investigate the effect of toe joint on the ankle kinetics. The ankle and toe joint were implemented by using series elastic actuators and they found that adding the toe joint could reduce the energy consumption in the prosthetic foot^10^. Honert et al. implemented the ankle and toe joint by using die springs and 1095 steel cantilever springs. They summarized that the toe stiffness had a lot of influence on the center-of-mass push-off dynamics, but the toe shape showed a slight effect on it^11^. Glanzer et al. developed the variable stiffness foot through the leaf-spring rollover structure. As a result, human subjects testing has shown that greater energy storage and return could be obtained with a lower stiffness setting^14^. All of these studies attempted to mimic the human gait behavior by implementing a human-like toe joint in the prosthetic foot.

**Figure 1:**
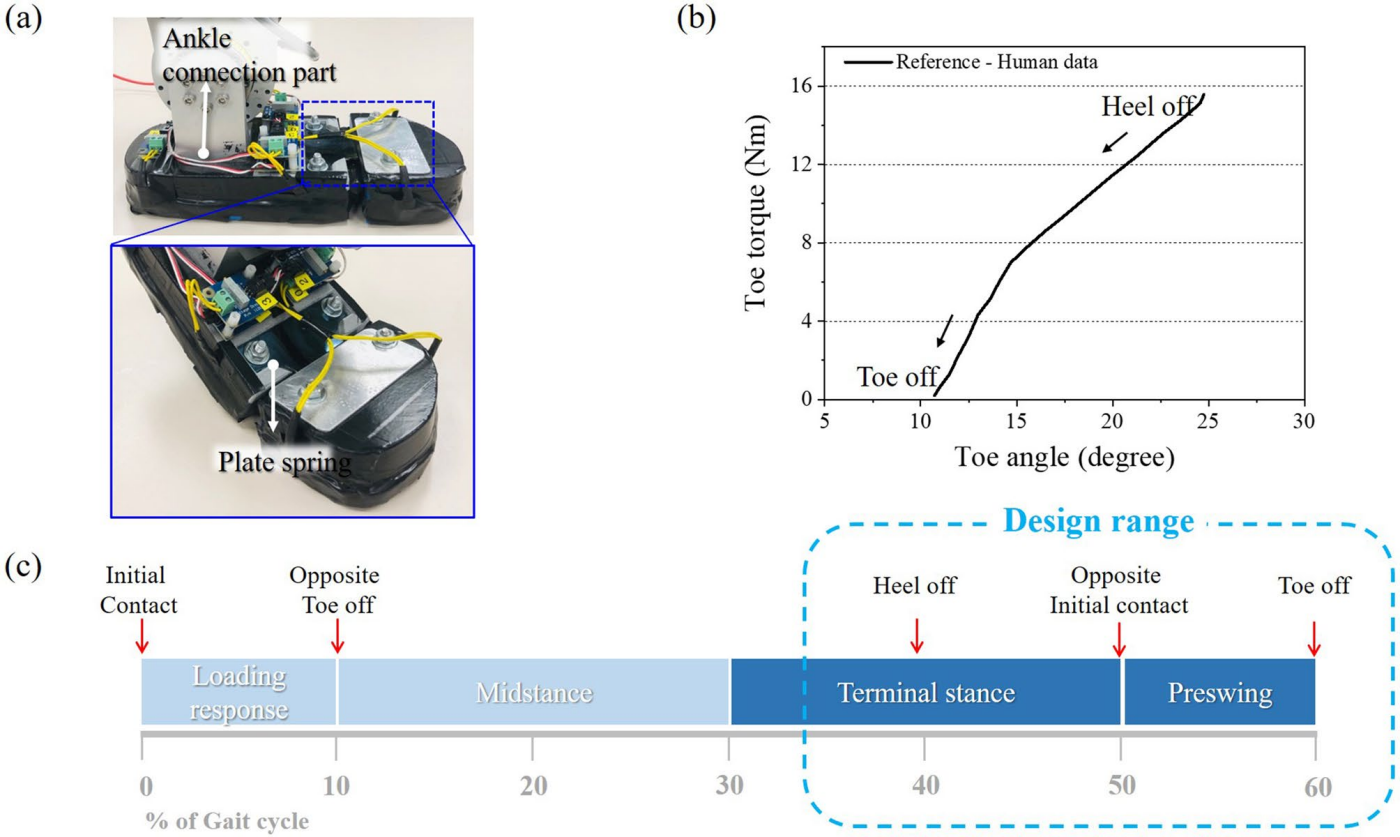
(**a**) A custom-built prosthetic foot which was used in previous study^16,17^; (**b**) human toe torque vs. angle graph from heel-off to toe-off^18^; (**c**) target design range of gait cycle in this study.

However, their designs were complex to be manufactured and had challenges implementing nonlinear stiffness characteristics. Also, they require additional (either active or passive) parts to implement the toe joint which is directly related to the weight increase^14,15^. To mimic the toe stiffness change of healthy human during the gait cycle (see Fig. 1b) without additional parts, having a different structural design should be considered for the toe joint. In addition, in the human foot, the toe angle changes by more than $15^{\circ }$ during toe-off. Considering this, a suitable bending structure should be considered to stably bend up to $20^{\circ }$ without any failure at toe-off^10,18^. Therefore, the shear and bending properties are important parameters to enable sufficient deformation of the prosthetic foot. The auxetic structure has drawn the focus due to its distinctive mechanical properties, such as increased shear resistance, energy absorption, and nonlinear behavior according to the deformation^19,20^. One challenge of applying such a designed structure to the prosthetic foot is that it is difficult to manufacture complex and curved shape with the conventional method. However, it can be overcome by using 3D printing technology, which has recently received interest in various fields and has an expanded range of shapes that can be manufactured^19^.

In this study, the toe joint behavior was investigated during terminal stance to improve walking performance as shown in Fig. 1c. We proposed the 3D printable foot structure (i.e., re-entrant and bending space and bending zone^21^) by using short carbon fiber reinforced polymer (onyx) to mimic human foot characteristics, especially the toe stiffness. The finite element analysis was conducted for the various toe joint geometries to simulate terminal stance from heel-off to toe-off. Then, to find the structure with characteristics similar to the human foot, the optimization was performed based on the toe joint geometries. Finally, simulation results such as toe/ankle kinematics, and COP were compared with human data to validate the FEA conditions.

**Figure 2:**
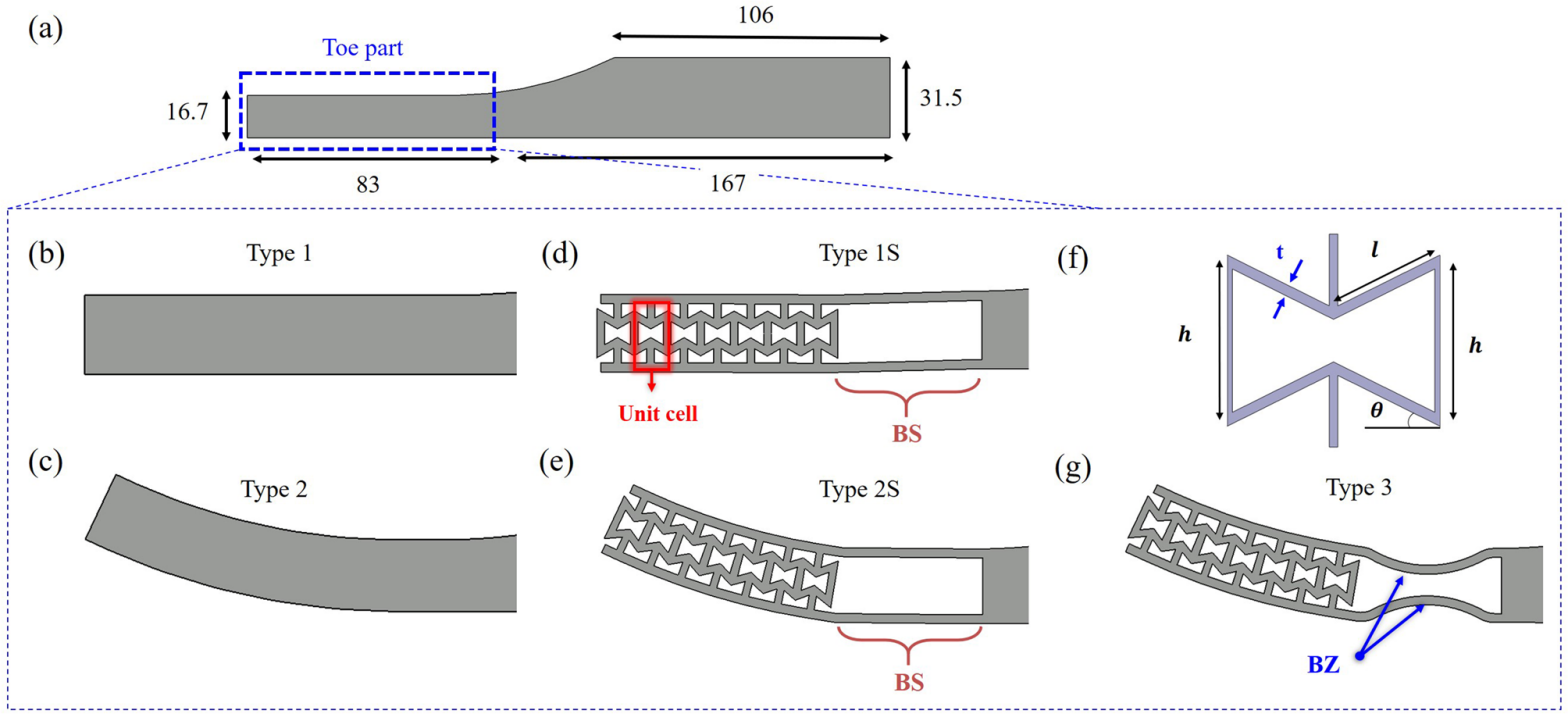
(**a**) Geometry and dimensions of prosthetic foot which was used in simulation. Various toe shape of prosthetic foot to implement toe joint to the prosthetic foot; (**b**,**c**) without structure and (**d**,**e**,**g**) with structure. (**f**) Geometry parameters of unit cell.

## Methods

### Toe joint design

The prosthetic foot geometry is shown in Fig. 2a. The total length of the foot was 250 mm and the height of the hindfoot was 31.5 mm. The length of the toe part was 83 mm, which was chosen based on the human factor considering where the forefoot strike occurs^22^. The dimensions were previously implemented to the foot of a custom-built powered prosthesis, AMPRO 2, shown in Fig. 1a^16,17^. To mimic human foot movement and toe characteristics during walking, the different toe shape was applied as shown in Fig. 2. In Fig. 2b, the original prosthetic foot has a flat rectangular shape, but it is improved by applying a curved shape to the forefoot to further mimic the human foot movement (see Fig. 2c). Also, in order to provide a more human-like toe joint, the prosthetic foot design was carried out by applying the re-entrant structure to the toe part as shown in Fig. 2d,e. Note that this structure has not only a negative Poisson’s ratio, but also it shows nonlinear compression behavior with increase stiffness, high energy absorption, high shear properties, and stable impact properties^19,20^. Therefore, it is suitable to use the re-entrant structure to prohibit the load concentration at toe tip, and to protect the part from impact loading. The unit cell of re-entrant structure and its four geometry parameters were depicted in Fig. 2f. These parameters were set to have a relative density of 0.4. The relative density of the re-entrant structure was defined by Eq. (1) as the ratio of the area of all lattice structures to the apparent area of the unit cell^20^.

1 $$\begin{aligned} \frac{A_{lattice}}{A} = \frac{t(h+2l)}{2l\cos {\theta }(h+l\sin {\theta )}} \end{aligned}$$

To avoid interference between the structures, the bending space (BS) was located behind the last unit cell (see Fig. 2d,e).

According to Zhu et al.^18^, human toe flexion reaches more than $20^{\circ }$ during toe-off. However, the original prosthetic foot near the BS is hard to endure this amount of bending since the stress is concentrated near the toe joint. Therefore, to enhance the bending characteristics of the forefoot, the bending zone (BZ) was considered as shown in Fig. 2g^21^. The BZ was designed with a curved structure on the inner and outer surfaces of the BS. It allows bending deformation to occur stably without unnecessary plastic deformation of the curved parts and material saving when it is manufactured.

**Figure 3:**
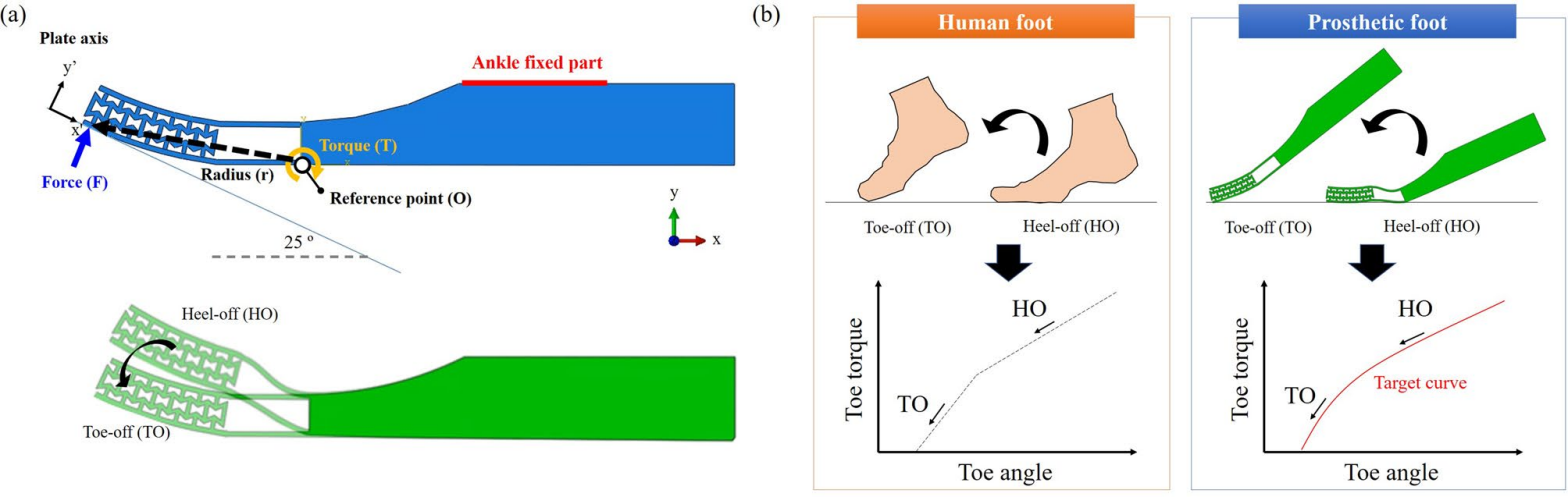
(**a**) Schematic of toe off simulation condition with prosthetic foot; (**b**) Simulation procedure during the HO–TO cycle and target stiffness curve compared with actual human walking.

### Finite element analysis

The mechanical behavior of the prosthetic foot during the stance phase was analyzed with the numerical simulation using commercial software ABAQUS/Standard(v6.14, ABAQUS Inc.). The prosthetic foot geometry was given in the simulation as Fig. 2a. The foot model was designed as a 2D surface model and meshed with plane strain elements CPE4R to reduce the computer run times^23,24^. For the simulation model, the mesh size was assigned to 0.6 mm so that three or more elements exist in the re-entrant structure^25^. For 3D printing, the onyx was considered which is produced by Markforged. Because Onyx is a short carbon fiber reinforced nylon filament, it has higher mechanical properties than the conventional plastic 3D filaments. Therefore, the tensile strength and the elastic modulus of the onyx were set to 38 MPa and 1.4 GPa, respectively. The simulation was conducted considering only the elastic properties, because it is assumed that the deformation occurs within the elastic region. During the gait cycle, repetitive deformation occurs in the prosthetic foot, and if deformation occurs in the plastic region beyond the elastic region, it cannot be recovered to its original shape. In this study, the terms heel-off (HO) and toe-off (TO) were newly defined in order to broadly deal with the behavior of the toe joint. HO means the state when the toe joint is completely flexed after the heel has already lifted off the ground. And TO means the state when the toe lifts off the ground after the push-off. In addition, a load criterion that the prosthetic foot can withstand in the predefined stance phase is also required. Therefore, the vertical ground reaction force during the human gait cycle was considered. Two maximum load peaks appear on the vertical ground reaction force graph during human walking. The first peak load is the impact force generated by the heel, and the second peak load represents the propulsion force that lifts the forefoot off the ground^26,27^. Therefore, considering the stance phase (i.e., from HO to TO), fracture or any failure should not occur in the designed prosthetic foot under the second peak load condition. In the case of an adult male with the weight of 100 kg, the second maximum load appears to be around 1000 N. This value is set as the load criterion for the foot design^27,28^. To simulate the stance phase of human gait, a rigid plate was placed at $25^{\circ }$ relative to the foot bottom as shown in Fig. 3. And the surface-to-surface contact constraint was applied between the rigid plate and foot bottom. The ankle fixed part was constrained not to move in any direction throughout the analysis. Also, the rigid plate was constrained in the x and y direction and fixed not to rotate in any direction by a coordinate system. Then, 1000 N was applied to the plate to deflect the foot in a normal direction to the plate.

The performance of the structures was evaluated by comparing the toe torque-angle graph of the simulation with that of the human data during toe-off^18^. The point, where the bending space ends, was set as the reference point (O) (see Fig. 3a), because the toe joint bending occurred at this point. Then, the toe torque was calculated using Eq. (2) as below^18,29^:

2 $$\begin{aligned} T = r\cdot F \end{aligned}$$

where T is the toe joint torque, r is the distance from the toe joint to O, and F is the reaction force of the prosthetic foot. And the angle between the toe tip and the foot bottom based on point O was defined as the toe angle. To calculate the toe angle, the position of the toe tip was derived from the simulation results.

**Figure 4:**
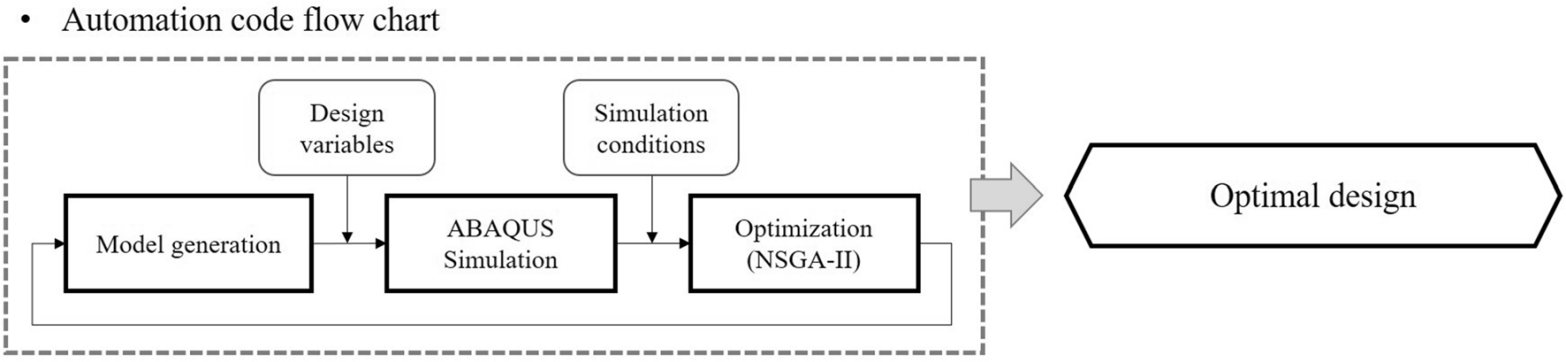
The flow chart of multi-objective optimization problem to design the prosthetic foot with optimal stiffness and maximum stress.

### Optimization method

The optimization algorithm was used to find an optimal structure of the prosthetic foot. The prosthetic foot should have variable stiffness, which is in accordance with human biomechanics as shown in Fig. 3b^18^. Also, when loading was applied to the prosthetic foot, the maximum stress should be less than the yield strength. Therefore, this is a multi-objective optimization design problem, which should consider two factors: stiffness and maximum stress. In this research, the non-dominated sorting genetic algorithm (NSGA-II), which is a classical genetic algorithm proposed by Deb et al. in 2002, was used for solving the given optimization problem^30,31^. This algorithm simultaneously optimizes each objective without being dominated by any other solution, unlike the single objective optimization algorithm.

The simulation was performed automatically by using MATLAB (v2020a, MathWorks Inc.) to proceed with the optimization. The flow chart of the optimization process is shown in Fig. 4. Design variables and simulation conditions were used as input data to MATLAB. A model was generated through the input design variables, and then ABAQUS simulation was performed through the input simulation conditions. After that, the simulation results were fed back to the input of the NSGA-II optimization algorithm, and the process was repeated until the solution converged to optimality.

## Results and discussion

### Geometry design for prosthetic foot

**Figure 5:**
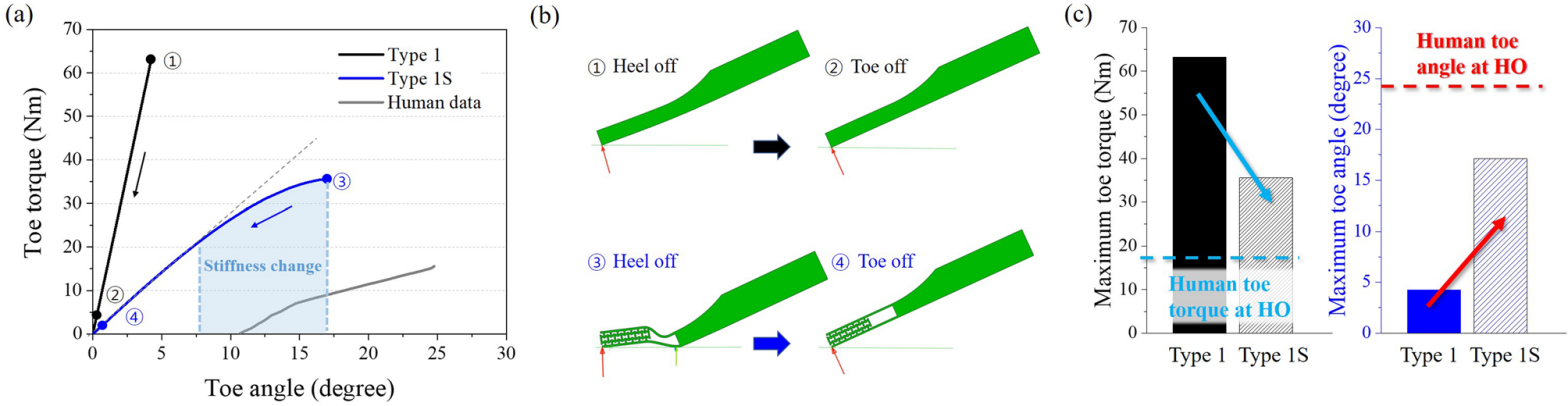
Simulation result of Type 1 and Type 1S models and reference human data^18^ from heel off to toe off. (**a**) Toe torque and angle graph. (**b**) Normal contact force position of Type 1 and 1S foot. (**c**) Maximum toe torque and angle.

To investigate the effect of the toe with the re-entrant structure on the toe characteristics during walking, the toe-off simulation was performed for Type 1 and Type 1S (Fig. 2b,d) and the results were depicted in Fig. 5. As a result of the toe-off simulation, Type 1 showed the linear behavior in the toe torque-angle graph in Fig. 5a. Since its cross-sectional shape was constant in a rectangular shape, there was no change in angular stiffness during toe-off. Also, the maximum toe torque value of Type 1 was 64 Nm ($\textcircled {1}$ in Fig. 5a) which is much higher than that of the human data (15.5 Nm)^18^. In addition, the maximum toe flexion was only $4.25^{\circ }$ which is about 5 times lower than that of humans ($24.75^{\circ }$) when the heel-off started^18^. This is because a large amount of the load is concentrated on the toe tip during the toe-off simulation, rather than moving from the center of the foot to the toe tip as shown in Fig. 5b. Therefore, the toe bending was not sufficiently performed, and the toe flexion angle was much lower than that of human. When the structure was applied to Type 1, the angular stiffness change occurred between $8^{\circ }$ and $17^{\circ }$ (see Fig. 5a) of the toe angles, which increased slightly. Some reaction force was acted at the center of the foot when the heel-off started, but still, the load was mainly concentrated on the toe tip as shown in Fig. 5b.

**Figure 6:**
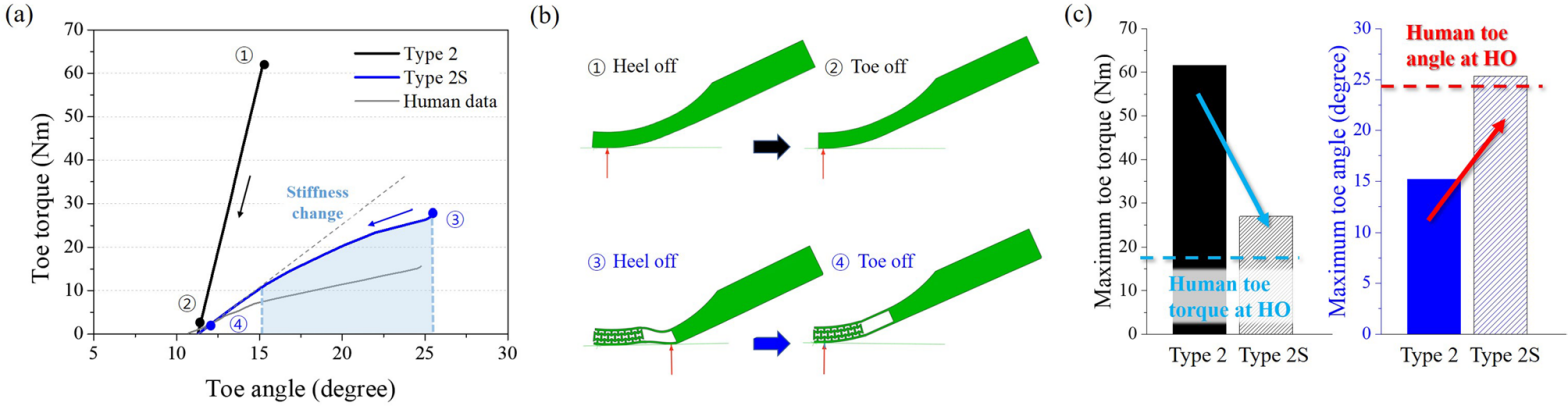
Simulation result of Type 2 and Type 2S models and reference human data^18^ from heel off to toe off. (**a**) Toe torque and angle graph. (**b**) Normal contact force position of Type 2 and 2S foot. (**c**) Maximum toe torque and angle.

Compared to Type 1, the maximum toe flexion angle was increased by 4 times, and the maximum toe torque was reduced by almost half ($\textcircled {3}$ in Fig. 5a). This showed that the toe behavior was improved when the re-entrant structure and bending space were applied. Nevertheless, the torque value was still high than that of the general human walking. Also, when the toe-off started, the toe angle was zero in both Type 1 and Type 1S cases ($\textcircled {2}$, $\textcircled {4}$ in Fig. 5a). This corresponds to the toe-off in actual human walking, where the toe flexion angle is about $10^{\circ }$ as shown in Fig. 5a. To mimic the natural human walking and to continuously move the loading point from the center of the foot to the toe tip, the curved shape was applied (e.g., Type 2 and Type 2S). Figure 6 shows the result of the simulation for Type 2 and Type 2S. Type 2 still showed a linear behavior in the toe torque-angle curve due to its constant geometry, while Type 2S showed angular stiffness change at about $15^{\circ }$ of toe angle. In both cases, the toe angle was $11.3^{\circ }$ when the toe-off finished ($\textcircled {2}$, $\textcircled {4}$ in Fig. 6a), which is more similar to human walking. Furthermore, in the case of Type 2S, the load point moved from the center of the foot to the toe tip, but for Type 2, it moved only 5 mm from the toe tip as shown in Fig. 6b. Therefore, Type 2S showed more similar behavior to the human gait. The maximum toe torque of Type 2 and Type 2S foot also decreased compared to Type 1 and Type 1S foot as shown in Figs. 5c and 6c. Especially, Type 2S showed the lowest toe torque among other cases. The maximum toe angle of Type 2S was about $25^{\circ }$, which was similar to human data as represented in Fig. 6c. As a result, the curved shape with re-entrant structure and bending space showed improved toe behavior during the stance phase (i.e., heel-off to toe-off).

### Bending zone design

**Figure 7:**
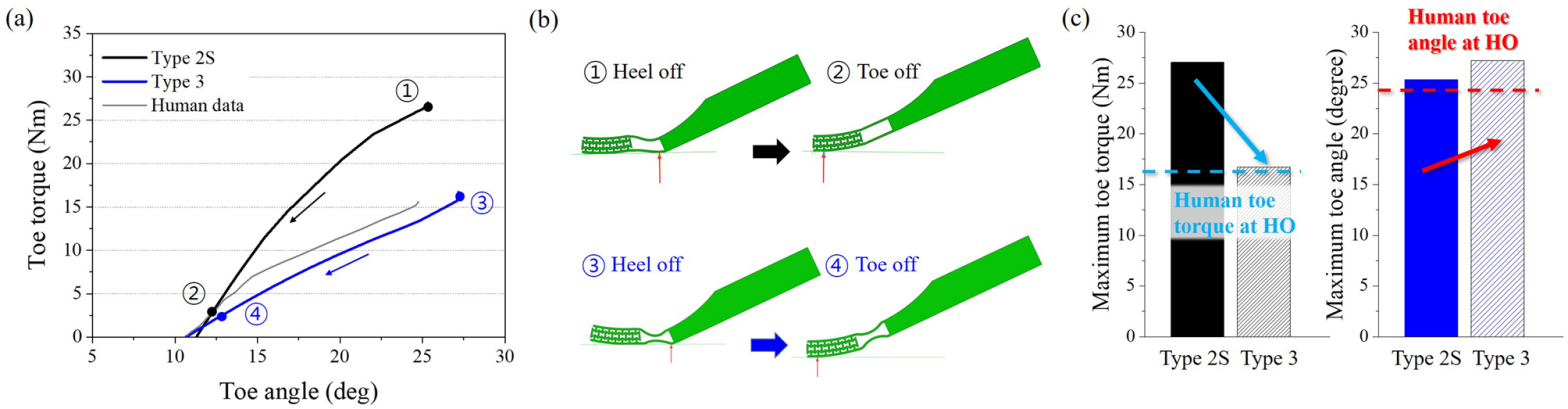
Simulation result of Type 2S and Type 3 models and reference human data^18^ from heel off to toe off. (**a**) Toe torque and angle graph. (**b**) Normal contact force position of Type 2S and 3 foot. (**c**) Maximum toe torque and angle.

The toe torque of the curved foot is still high compared with the human foot. This is because the current shape of the bending space has a limitation of the bending deformation as mentioned before. Therefore, to enhance the bending characteristics of the foot, the BZ was adapted in the bending space of Type 2S, resulting in Type 3 (see Fig. 2g). The simulation results of both types were compared as shown in Fig. 7. The toe torque was greatly reduced in Type 3 as shown in Fig. 7a. Also, the load point moved well from the center of the foot to the toe tip as shown in Fig. 7b.

To visualize this numerically, the maximum toe toque and the toe angle were derived from the torque-angle graph and compared with the result of the curved structured toe as shown in Fig. 7c. The toe torque was greatly reduced in Type 3, which was similar to the human data (15.5 Nm). In addition, the maximum toe angle was slightly increased after adapting the BZ. This is because the curvature (inner and outer) of the bending zone allows tensile and compressive deformations to occur more when the structure is bent into a curved shape^21^. Also, the curvature of the bending zone reduces unnecessary plastic deformation and structural damage. That resulted in the decrease of maximum stress and torque loaded on the structure.

### Optimization

**Figure 8:**
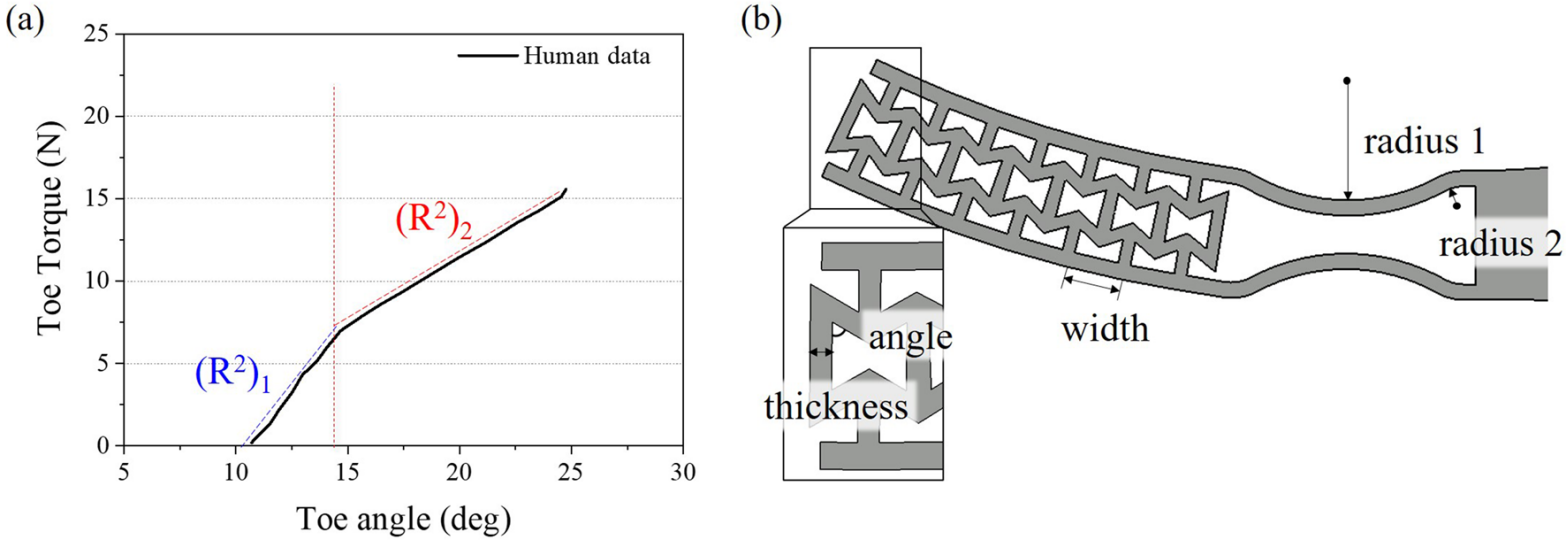
(**a**) Calculation area of coefficients of determination ($(R^{2})_{1}$ and $(R^{2})_{2}$) among objective functions; (**b**) design variables of the Type 3.

Although the characteristics of the current design are still far from that of the human foot (see Fig. 7a), the results shown above confirmed that the current design may have similar values (such as maximum toe torque and toe angle) to the human foot by modifying the dimensions of the structures. To find a structure that performs similarly to the human foot, an optimization process was conducted based on the Type 3 model. To do this, it is necessary to establish the standard indicating how well the stiffness characteristics match the human foot behavior. Therefore, the coefficient of determination ($R^{2}$) of the torque-angle curve in the simulation, which represents the difference between two curves, was derived based on the torque-angle curve of the human foot^32,33^. As shown in Fig. 8a, the slope of toe torque-angle curve changes rapidly emanating from $15^{\circ }$. To reflect this behavior appropriately, two $R^{2}$ values (i.e., $(R^{2})_{1}$ and $(R^{2})_{2}$) from linear regression were examined from each of two sections for the goodness of fit.

**Figure 9:**
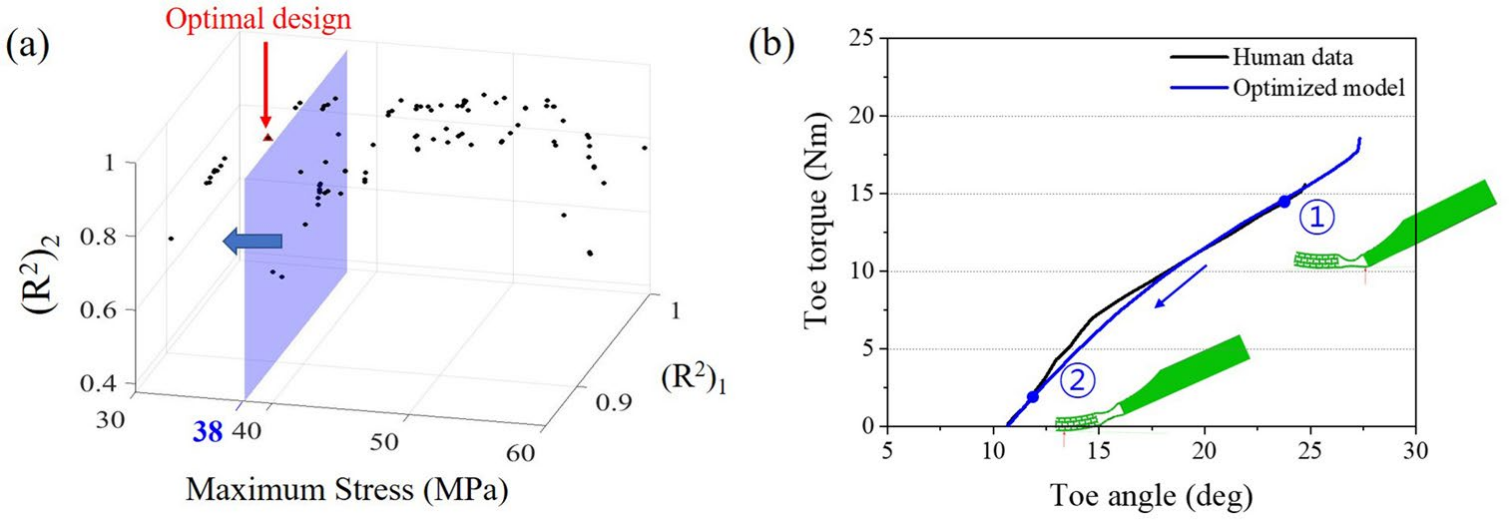
The results of the optimization process; (**a**) Pareto optimal solutions; (**b**) toe torque, angle graph and normal contact force position derived from heel off to toe off simulation.

Since the structure in the toe joint should have nonlinear stiffness characteristics without exceeding the yield strength, the objective functions were set as minimizing maximum stress loaded on the foot and maximizing $R^{2}$ values. The dimensions of the re-entrant structure (angle, thickness, and width) and bending zone (radius 1 and radius 2) were set as the design variables of the optimization as shown in Fig. 8b. Considering the dimensions of AMPRO2, the height of the re-entrant structure was fixed to make it lower than the ankle joint. The generation was set as 10 and the population as 95. The Pareto optimal solutions were derived from the optimization as shown in Fig. 9a. Among them, optimal solutions with a maximum stress of less than 38 MPa were selected, since the yield strength of the onyx is 38 MPa. From optimal solutions, an optimal solution model with high values of $(R^{2})_{1}$, $(R^{2})_{2}$ was selected. The values of the design variables for the selected solution were $67.04^{\circ }$ for the angle, 1.63 mm for the thickness, 5.91 mm for the width, 20.46 mm for the radius 1, and 45.60 mm for the radius 2. Consequently, the maximum stress of the structure was 36.59 MPa, the $(R^{2})_{1}$ value was 0.92, and the $(R^{2})_{2}$ value was 0.94. Generally, if the value of the coefficient of determination is more than 0.9, it can be considered that the two curves are considerably identical^32^. In addition, it was confirmed that the toe torque-angle curve of the optimized design has an almost similar curve to the human foot and the load point moved well from the center of the foot to the toe tip as shown in Fig. 9b. This result showed that the design of the prosthetic foot with the characteristics of the human foot is possible.

**Figure 10:**
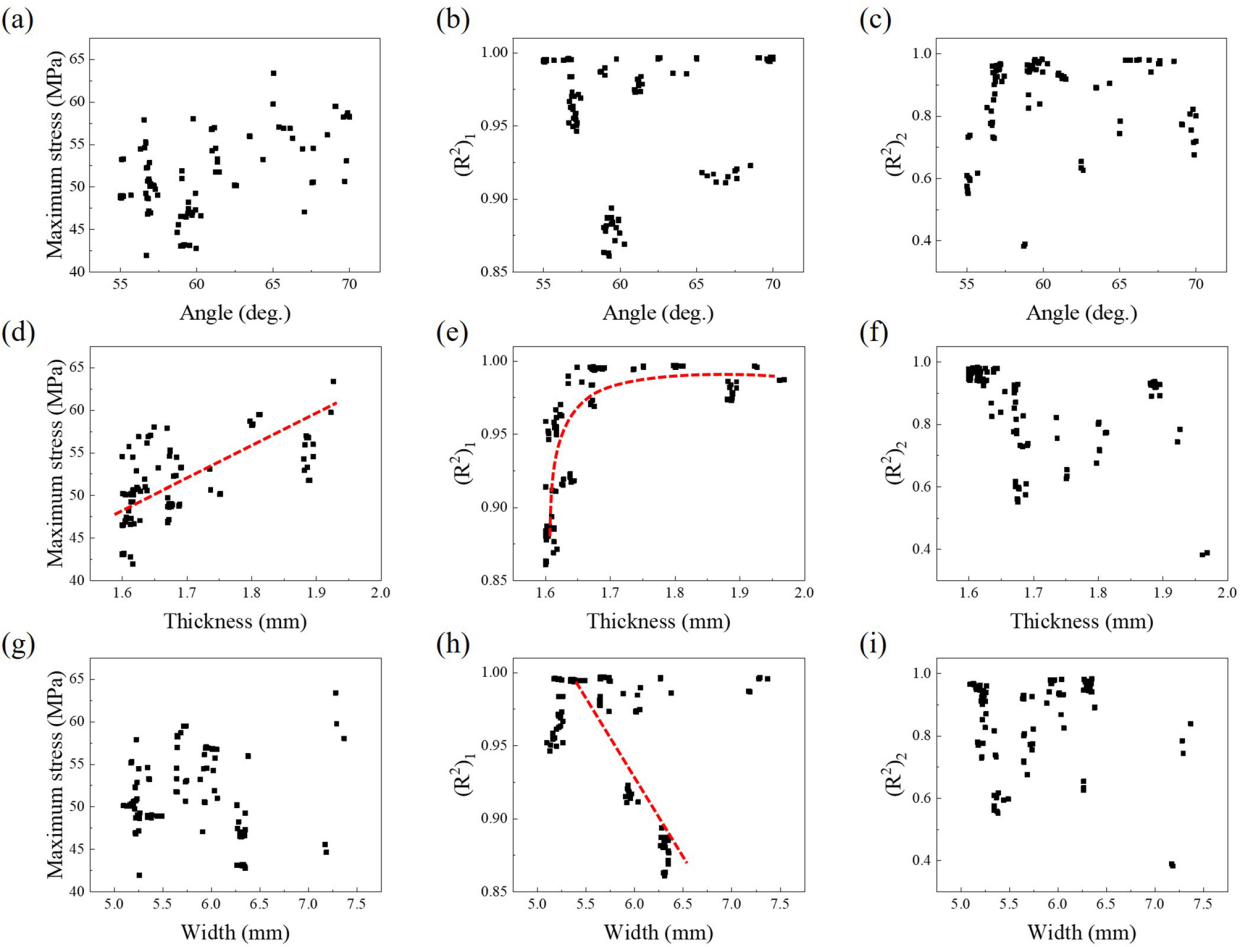
The objective functions (maximum stress, $(R^{2})_{1}$ and $(R^{2})_{2}$) results according to design variables of the re-entrant structure (angle, thickness, and width).

**Figure 11:**
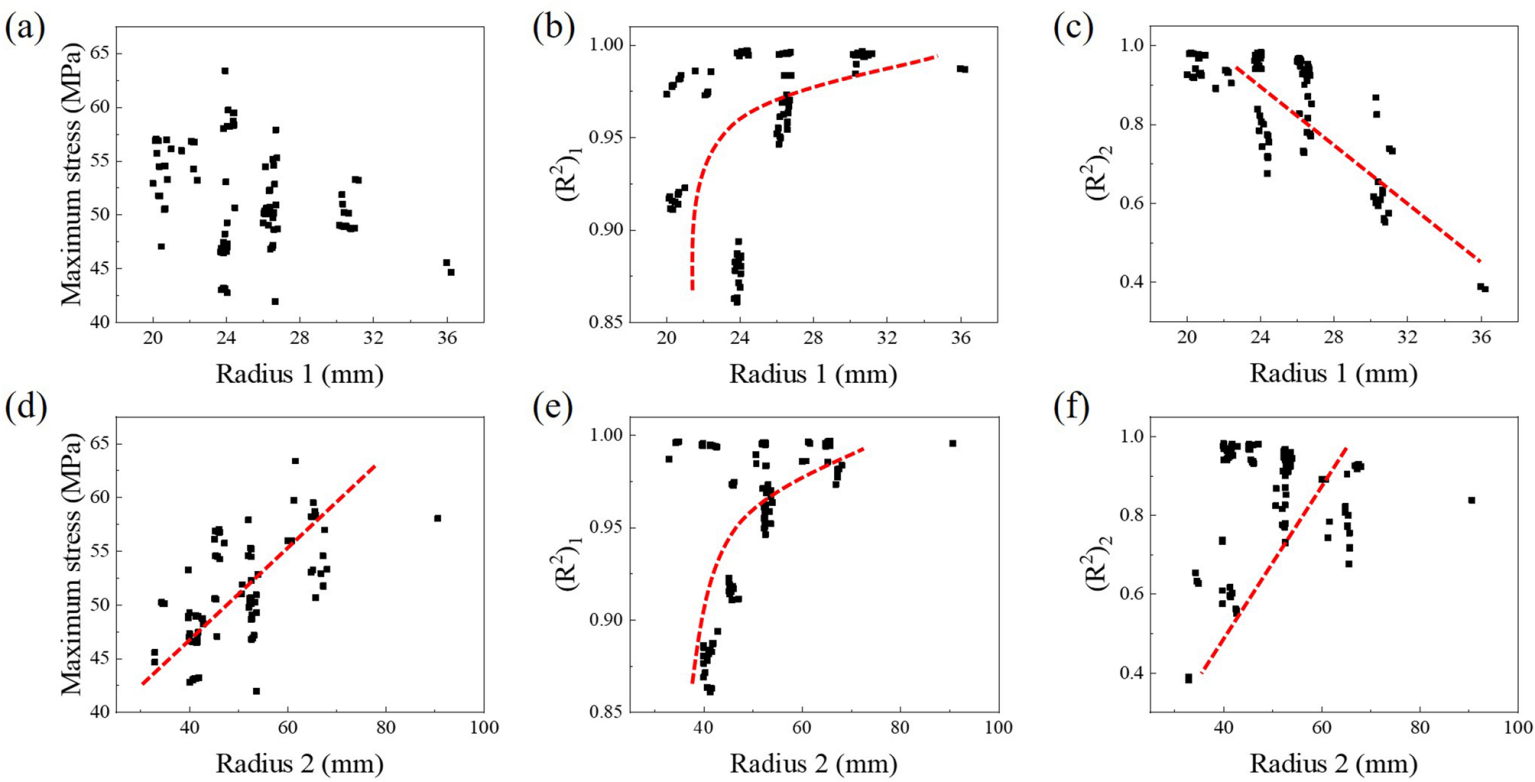
The objective functions (maximum stress, $(R^{2})_{1}$ and $(R^{2})_{2}$) results according to design variables of the bending zone (radius 1 and radius 2).

To analyze the effect of the design variables, object function results according to the design variables were plotted from the Pareto optimal solutions: Fig. 10 for the re-entrant structure (angle, thickness, and width), and Fig. 11 for the BZ (radius 1 and radius 2). It was confirmed that there was a nonlinear trend overall since each design variable is intricately entangled. Nevertheless, trends could be observed in certain variables. Among the variables for the re-entrant structure (see Fig. 10), the thickness and the width had a relationship with the $(R^{2})_{1}$ value, which affects the toe joint properties during the toe-off. Unlike this, no trend was found in the $(R^{2})_{2}$ value, and it means the thickness and the width have little effect on the toe joint properties during the heel-off. In addition, it could be found that selecting an appropriate thickness is necessary in order to lower the maximum stress. In the variables for the BZ (see Fig. 11), a more obvious trend could be found than the trend of the variables for the re-entrant structure. Both the $(R^{2})_{1}$ and $(R^{2})_{2}$ had a relationship with the variables for the BZ.

Since the bending characteristics of the toe joint are mainly determined by the bending zone, the dimensions of the bending zone have more influence on the $R^{2}$ values.

Therefore, it is essential to select appropriate dimensions of the BZ to implement the nonlinear characteristics of the toe joint and to reduce maximum stress. Consequently, an appropriate design for the characteristics of the human foot was established based on the influence of each design variable from the optimization process.

### Simulation condition validation

**Figure 12:**
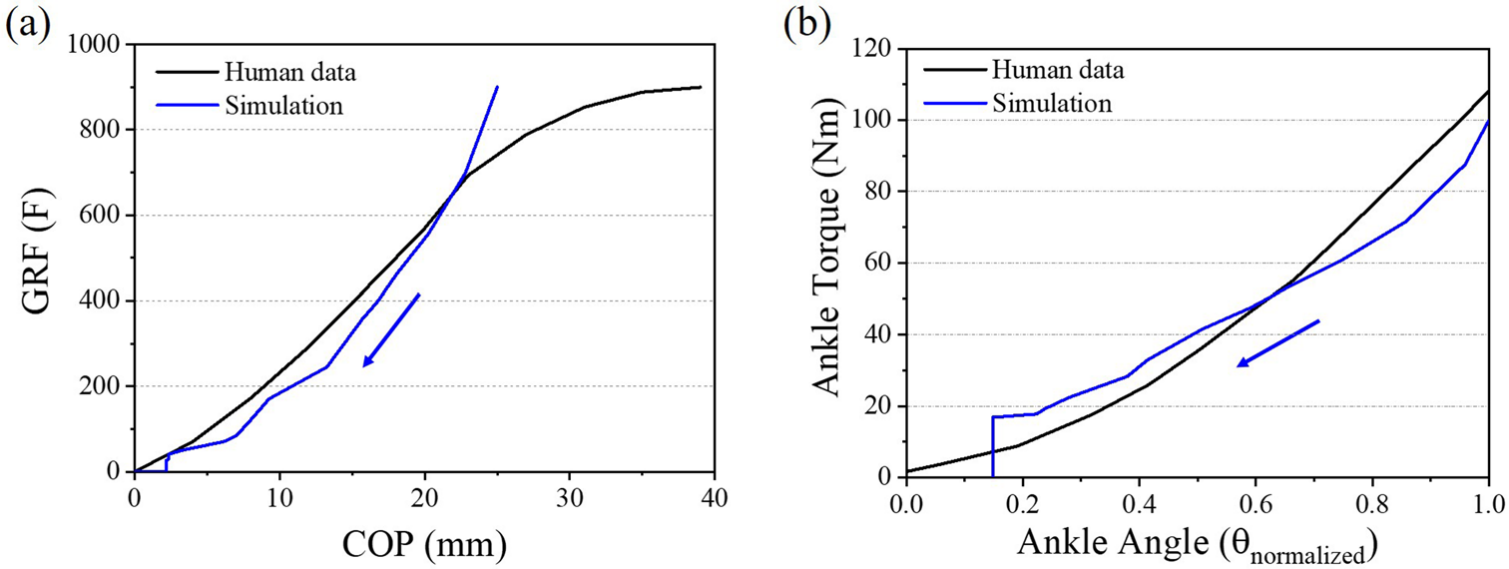
Comparison between human data and simulation result; (**a**) ground reaction force according to the center of pressure. (**b**) Normalized ankle angle according to the ankle torque.

So far, the simulation was conducted to analyze the toe characteristics in the stance phase. To simulate the human’s stance phase, the ankle part of the foot was constrained and the force was applied by a rigid plate assembled $25^{\circ }$ relative to the foot bottom. To validate these simulation conditions, the result of the simulation was compared to the human walking data. The reference data were used as human walking data such as ground reaction force (GRF), a center of pressure (COP), ankle angle, and ankle torque^18,34^. Firstly, the GRF according to the COP was analyzed between simulation results and human data. During the stance phase of human walking, the GRF started to decrease slowly when heel-off was performed, and then the GRF more rapidly decreased as the COP decreased as shown in Fig. 12a. Similarly, the simulation result shows that the GRF and COP decreased during the stance phase. However, in the simulation result, the slowly decreased behavior could not be found when the heel-off started. It is because the mid-stance and intact limb was not considered in this simulation where the share of mass is performed between the prosthetic limb and intact limb^35,36^. And, unlike human data, fluctuations in GRF values were found in the last period of the stance phase in the simulation result. This behavior is due to the gap between the re-entrant structure in the toe part. Nevertheless, it can be seen that the graph shows almost the same trend as shown in Fig. 12a. Secondly, the ankle kinematics was analyzed to validate the fixed condition of the ankle part in the simulation. When the push-off was performed during the stance phase of human gait, the ankle torque-angle decrease as shown in Fig. 12b. The simulation result showed similar behavior to the human gait and showed good agreement with the maximum torque of human data.

Because the ankle joint was not designed in this simulation, the exact ankle angle could not be directly compared to that of human. However, the tendency of the ankle torque-angle curves was similar based on the normalized ankle angle, and the maximum torque value showed good agreement with the human data. Therefore, it could be concluded that the loading and boundary conditions used in FEA were suitable for simulating human walking.

## Limitation and future work

The 3D printable foot structure of this study will be of great help in the rehabilitation treatment of patients. However, there are some limitations to this designed foot. First, the stiffness curve of the designed foot cannot be discrete. Since the designed foot was composed of a single part without additional joints, it is difficult to cause a rapid stiffness change. Due to this, the 3D printed foot shows continuous changes in stiffness unlike human foot with toe joint. Next, the user’s weight, who will use the 3D printed foot, could be limited. The currently designed foot has a stiffness that could only be applied to a specific weight range of patients. Therefore, in the case of patients who are out of the weight range, such as much lighter or heavier people, the dimension of the foot structure must be adjusted. Consequently, it was confirmed that the nonlinear behavior of the human toe joint can be implemented. Based on the result of this study, a designed prosthetic foot will be manufactured through 3D printing technology using the onyx filament. After that, we plan to verify the analysis results conducted in this study through an in-door experiment using the 3D printed prosthetic foot. In addition, it is necessary to design the heel part considering the shock absorption occurring in the heel strike, which is the starting stage of the stance phase. Therefore, in the end, it is planned to expand the design of the prosthetic foot that can stably mimic the nonlinear human gait behavior in the entire stance phase.

## Conclusion

In this study, the prosthetic foot design was conducted to improve gait performance similar to that of human walking. Then, the mechanical behavior of the designed foot was analyzed through FEA. The bending space with bending zone and the auxetic structure were applied to reduce the toe torque. As a result, the nonlinear toe stiffness behavior was implemented through these toe shape. Furthermore, toe deformation with more than $25^{\circ }$ could be achieved by a curved and structured toe shape. Also, the loading point was moved smoothly from the center of the foot to the toe tip. Based on the following results, optimization was conducted to find the optimal foot structure that implements human gait behavior. As a result, optimized foot structure was derived considering the complex effects of design variables. Compared with the human toe torque-angle data, the $(R^{2})_{1}$ value of the optimized structure was 0.92 and the $(R^{2})_{2}$ value was 0.94, which well mimics the stiffness characteristics of the human foot not exceeding the yield strength of the onyx. The simulation conditions used for toe-off was validated by comparing simulation result with human data. Consequently, the simulation result showed good agreement with human walking data in the GRF versus COP graph and ankle kinematics.
